# Beyond Unidirectional Decline: Identifying Nonlinear Frailty and Multimorbidity Trajectories in Older Adults

**DOI:** 10.1093/geroni/igaf122.1088

**Published:** 2025-12-31

**Authors:** Yanran Deng, Yan Luo, Yuming Chen, Zhou Yang, Beibei Xu

**Affiliations:** Peking University Health Science Center, Beijing, Beijing, China (People’s Republic); The University of Hong Kong, Hong Kong, China (People’s Republic); Peking University Health Science Center, Beijing, Beijing, China (People’s Republic); Peking University Cancer Hospital & Institute, Beijing, Beijing, China (People’s Republic); Peking University, Beijing, Beijing, China (People’s Republic)

## Abstract

Frailty and multimorbidity jointly drive adverse outcomes in older adults. However, their dynamic trajectories remain underexplored due to survival selection bias—where mortality systematically excludes high-risk individuals, distorting longitudinal progression patterns. This study aims to delineate joint trajectories of frailty and multimorbidity, and characterize their temporal synergies while accounting for the addressing selective survival effects. Using 12 waves (2011-2023) of the National Health and Aging Trends Study (NHATS), we annually assessed frailty (Fried phenotypic criteria) and multimorbidity (cumulative chronic conditions). To overcome survival selection bias inherent in aging cohorts, we extended group-based trajectory modeling through dual-process specification: a multivariate latent class model identifying progression typologies, coupled with a competing risks survival model. Shared parameters dynamically adjusted trajectory estimates for mortality-related attrition, ensuring unbiased characterization of temporal synergies. Among the 4,960 participants from the NHATS 2011 cohort included in this analysis, 2,931 (survey-weighted 57.2%) were female, and 2,070 died during follow-up (average annual attrition rate: 4.3%). Seven joint trajectories were identified, revealing three dominant patterns: stable phenotypes (Stable Low Risk, Persistent High Multimorbidity), progressive escalators (Late-Onset Frailty, Early Accelerated Frailty, Delayed Escalation), and nonlinear progressors (High Baseline Attenuation, Critical-State Reversal). Notably, Critical-State Reversal demonstrated frailty decline despite extreme multimorbidity progression, while Early Accelerated Frailty and Late-Onset Frailty diverged in progression timing despite comparable multimorbidity burdens, challenging assumptions of unidirectional frailty trajectories in aging. This study identifies seven distinct trajectories, and reveals unexpected heterogeneity in aging processes. These mortality-adjusted trajectories may offer a clinically actionable framework for risk stratification and personalized care planning.

